# FACTORS ASSOCIATED WITH THE AVAILABILITY OF SPECIALIZED RESOURCES TO TACKLE OBESITY IN HIGH MEDICAID NURSING HOMES

**DOI:** 10.1093/geroni/igac059.996

**Published:** 2022-12-20

**Authors:** Gregory Orewa, Ganisher Davlyatov, Rohit Pradhan, Justin Lord, Robert Weech-Maldonado

**Affiliations:** University of Alabama at Birmingham, Birmingham, Alabama, United States; University of Oklahoma, Oklahoma City, Oklahoma, United States; West Liberty University, West Liberty, West Virginia, United States; Louisiana State University at Shreveport, Bossier City, Louisiana, United States; University of Alabama at Birmingham, Birmingham, Alabama, United States

## Abstract

The purpose of this research is to explore the factors associated with the availability of specialized resources required to care for obese residents in high Medicaid nursing homes (NHs) (85% or higher). Due to the vagaries of payment models---Medicaid payments lag other modes of NH reimbursements—high Medicaid NHs typically report poorer quality and financial performance. Operating in a financial perilous environment, and with obesity among the elderly on the rise, high Medicaid NH may particularly struggle to obtain the appropriate resources essential to cater to obese residents' needs.Utilizing the resource-dependent theory, we hypothesized that occupancy rate, acuity index, and payer mix may be positively associated with the availability of obesity related specialized equipment in high Medicaid NHs. The study was conducted by merging survey and secondary data sources for the year 2017-2018. Obesity related data was collected via mail surveys sent to Directors of Nursing in high Medicaid NHs, The survey data was merged with the following secondary data sources: Brown University’s LTC Focus, Area Health Resource File, and the Medicare cost reports. The dependent variable was the summative obesity score that ranged from 0-19 with the larger number indicating greater availability of obesity-related equipment/services. An ordinary least square regression with propensity score weights (to adjust for potential non-response bias), and appropriate organizational/market level control variables were used for our analysis. Results suggest that payer mix (>Medicare residents) and acuity index were positively associated with the summative obesity score (p < 0.05). Policy and managerial implications are discussed.

